# Malaria burden and anti-malarial drug efficacy in Owando, northern Congo

**DOI:** 10.1186/s12936-015-1078-4

**Published:** 2016-01-08

**Authors:** Brice P. Singana, Hervé Bogreau, Brunelle D. Matondo, Louis R. Dossou-Yovo, Prisca N. Casimiro, Rigobert Mbouka, Kim Yen Ha Nguyen, Bruno Pradines, Leonardo K. Basco, Mathieu Ndounga

**Affiliations:** Unité de Recherche sur le Paludisme, Centre d’Etudes sur les Ressources Végétales (CERVE), BP 1249, Brazzaville, Republic of Congo; Unité de Parasitologie et d’Entomologie, Département des Maladies Infectieuses, Institut de Recherche Biomédicale des Armées, Brétigny-sur-Orge, France; Unité de Recherche sur les Maladies Infectieuses et Tropicales Emergentes (URMITE), UM 63, CNRS 7278, IRD 198, Inserm 1095, Aix-Marseille Université, Marseille, France; Centre National de Référence du Paludisme région Antilles-Guyane, Laboratoire de Parasitologie, Institut Pasteur de la Guyane, Cayenne, France; Direction interarmées du service de santé, Cayenne, France; Laboratoire National de Santé Publique (LNSP), BP 120 Brazzaville, Republic of Congo; Direction Départementale de la Santé (DDS) de la Cuvette, Owando, Republic of Congo; Centre National de Référence du Paludisme, Marseille, France

**Keywords:** Malaria, *Plasmodium falciparum*, Anti-malarial drug, Drug resistance, Microscopy, Rapid diagnostic test, Artemisinin, Combination therapy, Congo-Brazzaville

## Abstract

**Background:**

In the Republic of Congo, previous epidemiological studies have only been conducted in the south of the country where it is most accessible. Nationally representative data on the efficacy of new anti-malarial tools are lacking in the country. As an initial step to close the gap, clinical efficacy of two artemisinin-based combinations, artesunate-amodiaquine (ASAQ) and artemether-lumefantrine (AL), was assessed in Owando, a city in equatorial flooded forest in northern Republic of Congo.

**Methods:**

Under 12 years old febrile children attending public health facilities were screened for malaria parasites using lactate dehydrogenase (LDH)-based rapid diagnostic test (RDT) for malaria and microscopic examination of thick blood films. Patients with at least 1,000 asexual *Plasmodium falciparum* parasites*/*µl of blood were clinically examined, included after informed consent, and followed up for 28 days, according to the 2009 World Health Organization protocol. Patients were randomly assigned to co-formulated ASAQ (Coarsucam^®^) or AL (Coartem^®^) treatment groups. *Plasmodium falciparum* recrudescent isolates were compared to pre-treatment isolates by polymerase chain reaction (PCR) using *msp1*, *msp2*, and *glurp* genes to distinguish between re-infection and recrudescence.

**Results:**

Between November 2012 and February 2013, 857 under 12 years old febrile children were screened, of whom 198 (23.1 %) had positive RDT and 167 (19.5 %) positive thick films. ASAQ and AL efficacies were 92.7 and 94.2 % before PCR correction, respectively. After genotyping, the overall efficacy was 100 % for ASAQ and 98.0 % for AL.

**Conclusion:**

The data reported here represent partially the burden of malaria in 0–11 years old febrile children examined in public health centres of Owando city and serve as reference for further studies. Both artemisinin-based combinations were highly efficacious in patients under 12 years old with acute uncomplicated malaria. ASAQ was associated with more adverse events, which may reduce compliance in unsupervised treatment.

*Trial registration*: ACTRN12612000940875

## Background

Despite international commitments that have led to the global malaria strategy from which the first results are expected within the Millennium Development Goals in 2015 [1–4], the decline in malaria incidence in sub-Saharan African countries may be temporary. In 2013, it was estimated that 198 million malaria episodes occurred, of which 82 % (163 million) were in sub-Saharan African countries. Mortality remained at a high level with 584,000 deaths, of which 90 % were reported from sub-Saharan African countries [5]. Using malaria control tools, mainly long-lasting insecticide-treated nets (ITN), intermittent preventive treatment during pregnancy, and artemisinin-based combination therapy (ACT), some sub-Saharan African countries have registered a substantial reduction in malaria burden in recent years. The quality of health systems has also contributed to this positive trend. In Central African countries, however, the complexity of the ecological context and poorly operating health system, including the lack of malaria surveillance system, limit the implementation of effective strategy to control malaria.

After several decades of intensive use of chloroquine and sulfadoxine-pyrimethamine (SP) to treat uncomplicated malaria, these two classical anti-malarial drugs had become less effective [6–9], resulting in a high percentage of malaria cases seen in health centres [10] and an increase in the number of severe malaria cases and malaria-associated mortality in hospitals [11, 12]. As a consequence, in 2006, the Republic of Congo adopted ACT as the first-line treatment [13]. Two forms of ACT, artesunate-amodiaquine (ASAQ) and artemether-lumefantrine (AL), which have become first- and second-line anti-malarial treatment, respectively, have proven effective during the initial clinical assessment [14–16].

To encourage the use of these new treatments by health personnel, the Congolese government decreed in 2008 free malaria treatment for all children less than 15 years old. ITNs have also been distributed free of charge to pregnant women and children under 5 years of age. This mass distribution was later extended to the entire population and performed regularly. Pregnant women also receive three doses of SP for intermittent preventive treatment of malaria. These measures have contributed to the reduction of malaria in pregnant women and children [17]. However, the available data are still inadequate to reflect the evolution of malaria throughout the country since most previous studies had been conducted in southern Republic of Congo.

The present study was conducted to assess the therapeutic efficacy of ASAQ and AL from November 2012 to February 2013 in the city of Owando, located in equatorial flooded forest in northern Republic of Congo. All febrile children aged less than 12 years spontaneously consulting at one of two public health centres were screened for the presence of malaria parasites using rapid diagnostic test (RDT) and microscopy. The objectives of this study were to determine the burden of malaria among febrile children less than 12 years old consulting spontaneously at the health centres during the study period and to evaluate the tolerance and effectiveness of these two forms of ACT.

## Methods

### Study site

**Fig. 1:**
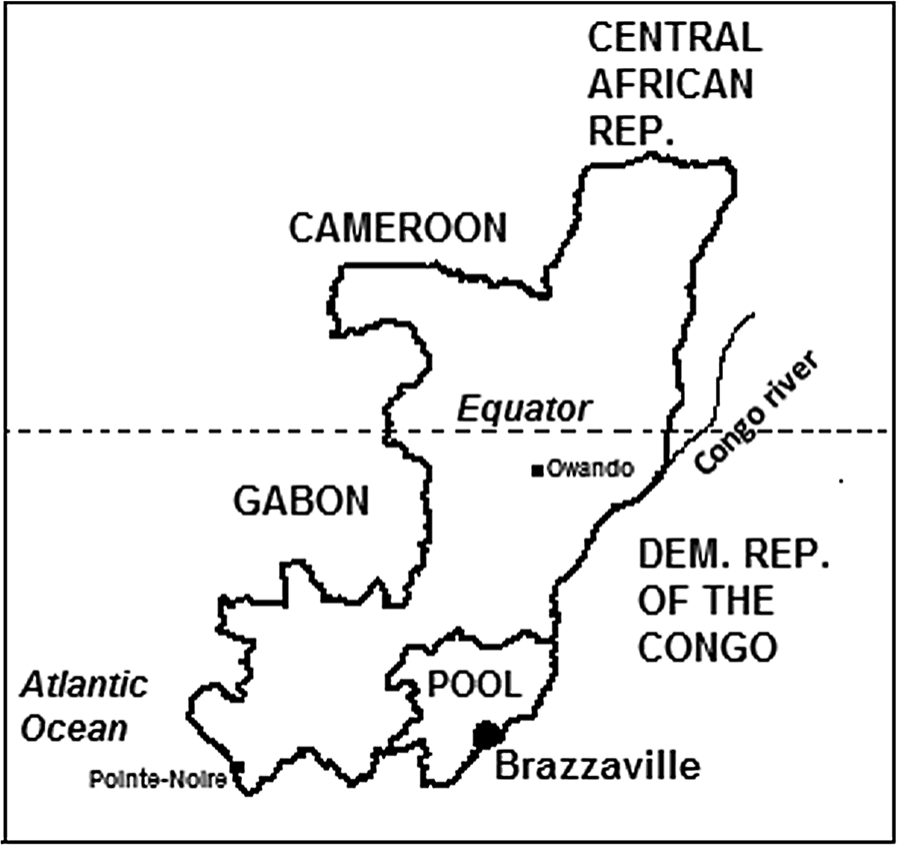
Map of the Republic of Congo showing the location of Owando city

Owando (latitude, 0°28′54″ South; longitude, 15°53′59″ East) is the main administrative city of Cuvette Department located 550 km to the north of Brazzaville, the capital city of the Republic of Congo, and 70 km to the south of the Equator, along the Kouyou River, one of the tributaries of the Congo River (Fig. 1). The climate is sub-equatorial with two rainy seasons, from October to December and April to May, and two dry seasons, from June to September and January to March. Owando is a town in flooded forest and is a strategic choke point for northern African countries and migrants from Cameroon and other African countries. Except for a study on the in vitro chemosusceptibility of *P. falciparum* [18], there are no existing data on malaria in this part of the country. The city has a general hospital, two public health centres (No. 1 and No. 2), a military clinic, and private health facilities. The present study was conducted in public health centres No. 1 and No. 2, which are separated by about 2 km.

### Malaria diagnosis and inclusion of children under 12 years old

Basic clinical information (age, weight, height, axillary temperature) of patients from 0 to 11 years was recorded in individual clinical forms. In the laboratory, fingerpick capillary blood was collected for RDT for malaria (Advantage Mal Card, J. Mitra & Co. Pvt. Ltd., New Delhi, India), preparation of thick films, haematocrit measurement, and storage of parasite DNA. Giemsa-stained thick films were examined under the microscope to determine the parasite density. Children with parasite density equal to or greater than 1000 asexual parasites/µl of blood were examined by a medical doctor. Children with fever (axillary temperature ≥37.5 °C), sufficient parasite load (≥1000 *P. falciparum* asexual parasites/µl), without any concomitant febrile illness or any signs of danger or severe and complicated malaria, were included according to the 2009 WHO protocol for the assessment of anti-malarial drug efficacy after informed consent [19].

### Treatment and follow-up

This was an open-label study. Eligible patients were allocated to one of the two treatment groups after randomization in blocks of 10 according to a pre-established list of treatment groups. Blocks 1, 3, 5, 7, and 9 were affected to ASAQ; patients assigned to blocks 2, 4, 6, 8, and 10 were treated with AL. Patients assigned to AL group (20 mg artemether + 120 mg lumefantrine, Coartem^®^; Novartis, Basel, Switzerland; lot F2545) received 1 tablet per dose for 5–14 kg body weight, 2 tablets per dose for 15–24 kg body weight, 3 tablets per dose for 25–34 kg body weight, and 4 tablets per dose for ≥35 kg body weight. Each patient received a total of 6 doses (first dose at inclusion on day 0, second dose 8 h after the first dose on day 0, then one dose in the morning and another dose in the evening on days 1 and 2). Patients treated with artesunate-amodiaquine (ASAQ, Coarsucam^®^, Sanofi-Aventis, Casablanca, Morocco; lot 5250) received one tablet containing 25 mg of AS/67.5 mg of AQ for <9 kg body weight, one tablet of 50 mg of AS/135 mg AQ for 9 to <18 kg, one tablet of 100 mg of AS/270 mg AQ for 18 to <36 kg, and two tablets of 100 mg of AS/270 mg AQ for ≥36 kg of body weight. ASAQ was administered once daily for 3 days. Both drugs were provided by Drug Resistance and Containment, Global Malaria Programme of the World Health Organization (WHO), Geneva, Switzerland.

The patients were followed on days 1, 2, 3, 7, 14, 21, and 28, as in the previous studies on the efficacy of ASAQ and AL in the Republic of Congo. Clinical examination and measurement of axillary temperature were performed during each visit, and any adverse events or unauthorized concomitant therapies were recorded. As recommended in the 2009 WHO protocol [19], blood films were examined on days 2, 3, 7, 14, 21, 28, and during any unscheduled visit if the patient became febrile. If parasites reappeared on or after day 7, fingerprick capillary blood was collected on Whatman^®^ 3MM filter paper for polymerase chain reaction (PCR) analysis. The filter papers were stored in airtight plastic bags with desiccant and stored at room temperature.

### DNA extraction and *Plasmodium falciparum* genotyping

In case of treatment failure, parasites before treatment (day 0 pre-treatment sample) were compared to parasites detected on the day of treatment failure. Parasite DNA was extracted from filter papers using EZNA blood DNA kit (Biofidal, Vaulx-en-Velin, France) according to the manufacturer’s recommendations. The allelic families and fragment size of merozoite surface antigen gene 1 (*msa1*, NCBI Gene ID: 813575, PlasmoDBlocus tag: PF3D7_0930300), merozoite surface antigen gene 2 (*msa2*, NCBIGene ID: 812660, PlasmoDBlocus tag: PF3D7_0206800), and the fragment size of glutamine-rich protein (*glurp,* NCBIGene ID: 810501, PlasmoDBlocus tag: PF3D7_1035300) were compared, as recommended by the WHO [20].

### Statistical analysis and treatment outcomes

#### Malaria burden

Clinical data from all enrolled children were entered into Excel spreadsheet. The analysis was performed using Epi-info 6.04 (Centres for Disease Control and Prevention, Atlanta, GA) to determine the percentage of febrile children attending public health facilities with malaria parasites detected with either microscopy or RDT. The performance of microscopy, as compared to RDT, was assessed by calculating the sensitivity (Se), specificity (Sp), positive predictive value (PPV), and negative predictive value (NPV). Cohen’s kappa coefficient (κ) was determined to measure the degree of agreement between the two diagnostic tests [21]. The 95 % confidence intervals [95 % CI] of the proportions of positive thick films and RDT, Se, Sp, PPV, and NPV were determined using the exact binomial method.

#### Sample size determination

The sample size was calculated based on an expected failure rate of 5, 10 % precision, and a confidence level of 95 %, with an estimated loss of 20 % due to exclusions, withdrawals, and loss-to-follow-up. At least 60 patients per treatment group were recruited [19].

#### Treatment outcomes

Clinical and parasitological data were analysed using the pre-programmed Excel spreadsheet provided by the Global Malaria Programme, WHO (Geneva, Switzerland). Patients who were excluded or lost to follow-up during the 28-day period were excluded from further analysis. Per protocol analysis is recommended by the WHO protocol and allows comparison of data with those of other sentinel sites in the country, mainly Brazzaville. Treatment responses were classified before and after PCR correction, as described in previous studies [15, 16].

The baseline characteristics of patients were compared using the Student’s *t* test. The percentage of early treatment failure (ETF), late clinical failure (LCF), late parasitological failure (LPF), and adequate clinical and parasitological response (ACPR) was calculated. Treatment failure rate was defined as the number of patients responding with ETF, LCF, or LPF divided by the total number of included patients who completed the 28-day follow-up. The 95 % CI of the outcomes (ETF, LCF, LPF and ACPR) was determined using the exact binomial method. Treatment outcomes before and after PCR correction were presented on Kaplan–Meier survival curves and compared using the LogRank test. The percentages of patients responding with ACPR and the frequencies of adverse effects associated with each ACT were compared using the Fisher’s exact test.

## Results

### Malaria burden in 0–11 years old children

**Fig. 2:**
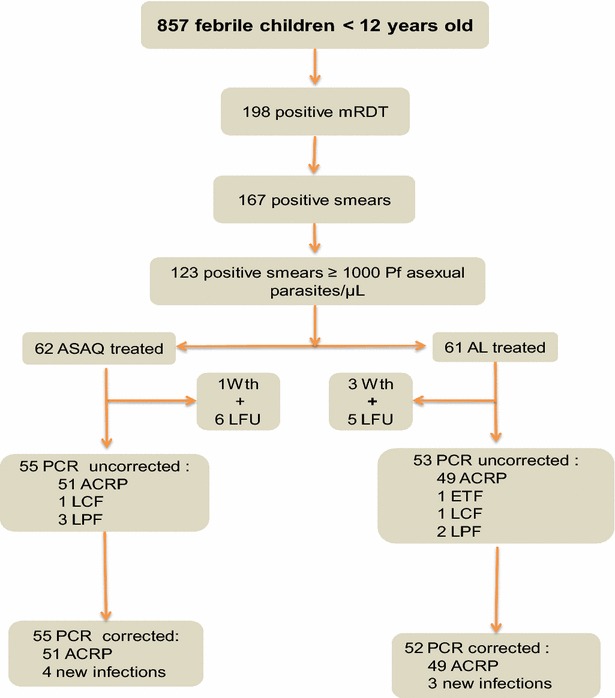
Enrolment and flow diagram. *mRDT* malaria rapid diagnostic test, *Pf* *P. falciparum*, *ASAQ* artesunate-amodiaquine, *AL* artemether-lumefantrine, *Wth* withdrawn, *LFU* lost to follow up, *ETF* early treatment failure, *LCF* late clinical failure, *LPF* late parasitological failure, *ACRP* adequate clinical and parasitological response

**Fig. 3:**
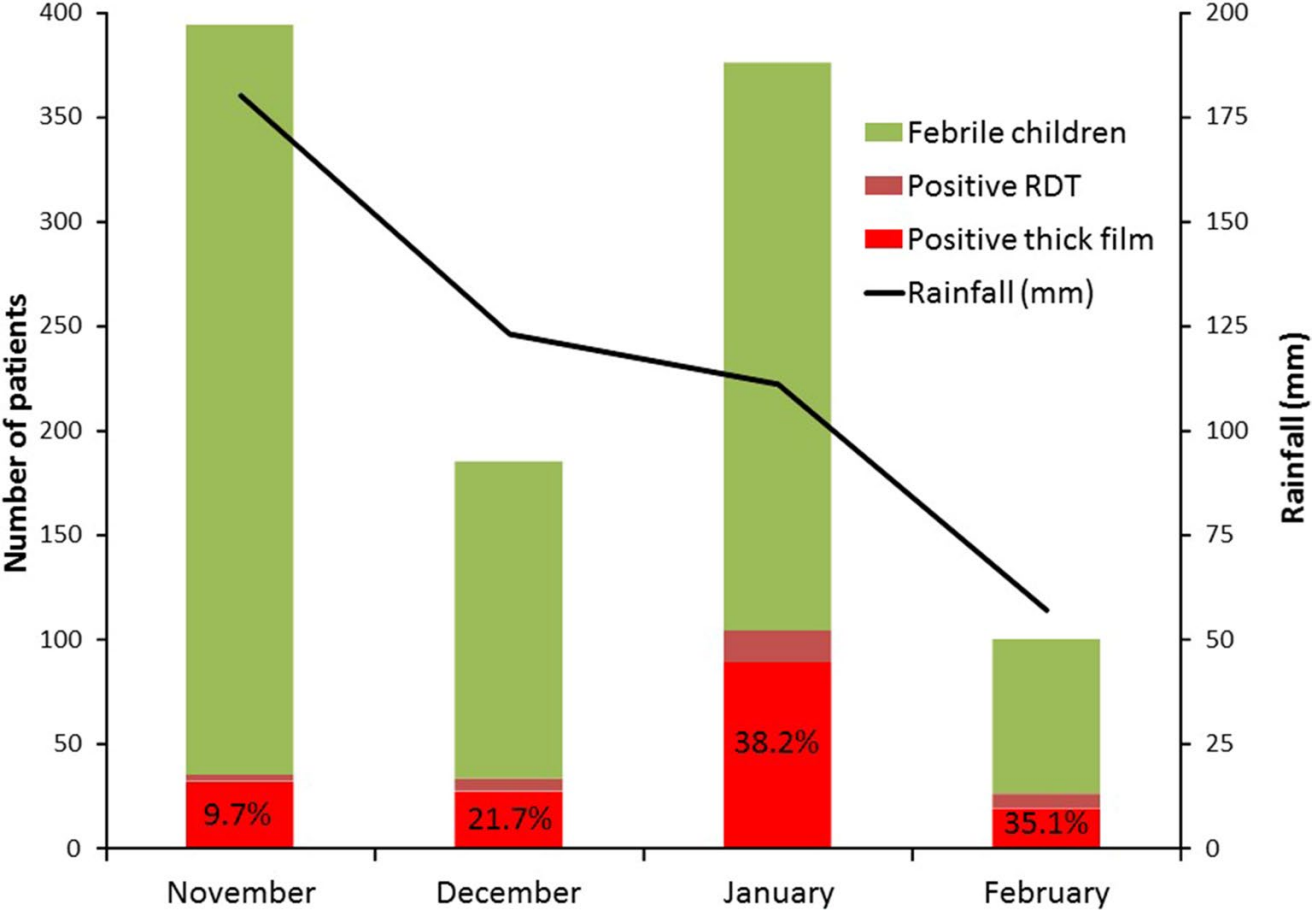
Malaria burden among febrile children less than 12 years old consulting at the health centres in Owando (northern Republic of Congo) from November 2012 to February 2013. Malaria-associated febrile cases are expressed as the percentage of *Plasmodium falciparum* infections confirmed by RDT (*Positive RDT*) and microscopy (*Positive thick film*) at the bottom of the *bar*

The study was conducted from November 2012 to February 2013. A total of 857 febrile children aged from 0 to 11 years were screened for malaria parasites (Fig. 2). Among these children, 198 (23.1 %) had positive RDT, and 167 (19.5 %) had positive thick blood films (Fig. 3; Table 1). Of 198 positive RDT, 167 (84.3 %) were RDT positive and microscopy positive, while 31 (15.7 %) had negative thick blood films. When compared to microscopy results, the sensitivity and specificity of Advantage Mal Card^®^ were 100 and 95.5 %, respectively. The kappa coefficient (κ) was 0.88, i.e. there was an excellent concordance between RDT and microscopy results.

**Table 1:** Performance of rapid diagnostic test for malaria (Advantage Mal Card^®^) compared to microscopy in health facilities in Owando, Republic of Congo

RDT result	Microscopy (n)	
	Positive	Negative
Positive	167	31
Negative	0	659

Sensitivity, 100 % (95 % confidence interval [95 % CI] 97.8–100 %); specificity, 95.5 % (95 % CI 93.7–96.9 %); positive predictive value, 84.3 % (95 % CI 78.5–89.1 %); negative predictive value, 100 % (95 % CI 99.4–100 %)

*RDT* rapid diagnostic test

The percentage of malaria cases increased when rainfall decreased (Fig. 3). In November 2012 (monthly rainfall, 180 mm), when the largest number of febrile children were examined in the present study, only 32 of 359 (8.9 %) thick films and 35 of 359 (9.7 %) RDT were positive. In December 2012 (monthly rainfall, 123 mm), 27 of 152 (17.8 %) thick films and 33 of 152 (21.7 %) RDT were positive. High percentage of febrile patients with malaria parasites was registered when rainfall decreased (monthly rainfall, 111 mm in January 2013 and 57 mm in February 2013): in January 2013, 89 of 272 (32.7 %) patients with positive microscopy and 104 of 272 (38.2 %) positive RDT and in February 2013, 19 of 74 (25.7 %) patients with positive microscopy and 26 of 74 (35.1 %) patients with positive RDT.

### Drug efficacy

#### Enrolled patients

From November 2012 to January 2013, 123 malaria-infected symptomatic children were randomly assigned to ASAQ (62 children) or AL (61 children) treatment group (Fig. 2). The baseline characteristics of both groups were comparable, except for age (Table 2). In the AL treatment group, there were more children aged less than 5 years.

**Table 2:** Baseline characteristics of febrile children with uncomplicated falciparum malaria enrolled in artesunate-amodiaquine (ASAQ) and artemether-lumefantrine (AL) groups

Characteristics	Treatment group	
	ASAQ	AL
No. of included patients	62	61
Age (months), mean ± SD (range)	71.0 ± 31.8 (9–132)	57.3 ± 30.4 (6–132)
<60 months (<5 years): N; % (95 % CI)	19; 30.6 (19.6–43.7)	29; 47.5 (34.6–60.7)
60–132 months (5–11 years): N; % (95 % CI)	43; 69.4 (56.3–80.4)	32; 52.5 (39.3–65.4)
Weight (kg), mean ± SD (range)	19.9 ± 7.8 (9–57)	18.1 ± 7.8 (7–44)
Sex ratio (F/M)	0.94 (30/32)	1.26 (34/27)
Axillary temperature, mean ± SD (range) (°C)	37.9 ± 1.0 (36.0–40.0)	38.0 ± 1.0 (36.0–40.4)
Parasite density (asexual parasites/µL)		
Geometric mean (range)	37,600 (1550–955,000)	34,200 (1000–700,000)
Self-medication before consultation: N; % (95 % CI)	0; 0 (0–5.8)	3; 4.9 (1.0–13.7)
Haematocrit, mean ± SD (%)	32.9 ± 5.0	31.9 ± 5.4

There was a significant difference (p = 0.01) in age between the two treatment groups

*SD* standard deviation, *95* *% CI* 95 % confidence interval

Among ASAQ-treated children, one child was referred to the general hospital for extreme fatigue and was excluded on day 1, and six were lost to follow-up: one patient on day 14, three patients on day 21, and two patients on day 28. On day 1, 2 of these 6 patients lost-to-follow-up were still febrile, whereas on day 2 and day 3 all of them were afebrile. On day 2, 6 of 61 (9.8 %) patients treated with ASAQ still presented low parasitaemia. On day 3, all patients had negative smears.

In AL-treated group, three patients were excluded after enrollment: two patients were not infected with malaria parasites on day 0 after controlling their thick films; 1 patient had repeated vomiting and was unable to swallow oral medication on day 1. Six AL-treated patients were lost to follow-up. On day 1, 11 of 56 (19.6 %) patients (per protocol population) were still febrile. On day 2 and day 3, all patients were afebrile. On day 2 and day 3, 7 (12.5 %) and 1 (1.8 %) patients had positive thick films, respectively. On day 7, all patients had negative microscopy results.

#### Treatment outcomes

**Fig. 4:**
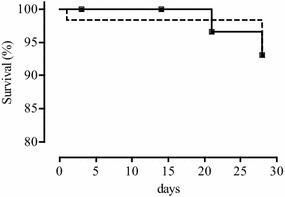
Kaplan–Meier survival analysis of PCR-uncorrected outcomes of ASAQ (*black squares*) and AL (*black circles*) treatment

**Fig. 5:**
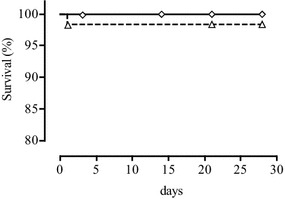
Kaplan–Meier survival analysis of PCR-corrected outcomes of ASAQ (*white diamonds*) and AL (*white triangles*) treatment

Per protocol analysis of the ASAQ treatment group showed four cases of treatment failure: 1 of 55 (1.8 %) patients presented LCF and 3 (5.5 %), LPF (Table 3). Genotyping showed that all four treatment failures were due to new infections, and these patients were censured. The overall efficacy represented by ACPR rate was 100 %. Among 52 eligible AL-treated patients (per protocol population), one (1.9 %) patient hospitalized during the night from day 0 to day 1 was considered as ETF; one (1.9 %) responded with LCF, and two (3.9 %) with LPF. After PCR analysis, the reappearance of parasites in patients with LCF and LPF was shown to be due to new infections, and these patients were censured. The PCR-corrected ACPR rate of AL was 98.0 %. The PCR corrected data of both treatments in the per-protocol analysis showed no significant difference in efficacy (p = 0.5). LogRank survival analysis of Kaplan–Meier curves did not show any statistically significant difference (P > 0.05) between the efficacy of ASAQ and AL before and after PCR correction (Figs. 4, 5).

**Table 3:** Artesunate–amodiaquine and artemether-lumefantrine efficacy on day 28 in Owando, Republic of Congo

	ASAQ	AL
No. of included patients (intention-to-treat)	62	61
PCR-uncorrected responses on day 28		
Withdrawn or lost to follow up: N; % (95 % CI)	7; 11.3 (4.7–21.9)	9; 14.8 (7.0–26.2)
Per protocol population: N; % (95 % CI)	55; 88.7 (78.1–95.3)	52; 85.2 (73.8–93.0)
Failures: N; % (95 % CI)	4; 7.3 (2.0–17.6)	4; 7.7 (2.1–18.5)
ETF: N; % (95 % CI)	0; 0 (0–5.8)	1; 1.9 (0–10.3)
LCF: N; % (95 % CI)	1; 1.8 (0–9.7)	1; 1.9 (0–10.3)
LPF : N; % (95 % CI)	3; 5.5 (1.1–15.1)	2; 3.8 (0.5–13.2)
ACPR: N; % (95 % CI)	51; 92.7 (82.4–98.0)	48; 92.3 (81.5–97.9)
PCR-corrected responses on day 28		
Withdrawn + lost-to-follow-up + censured due to new infections: N; % (95 % CI)	11; 17.7 (9.2–29.5)	12; 19.7 (10.6–31.8)
Per protocol population: N; % (95 % CI)	51; 82.3 (70.5–90.8)	49; 80.3 (68.2–89.4)
ETF: N; % (95 % CI)	0; 0 (0–7.0)	1; 2.0 (0.1–10.9)
Recrudescence: N; % (95 % CI)	0; 0 (0–7.0)	0; 0 (0–7.2)
ACPR: N; % (95 % CI)	51; 100 (93.0–100)	48; 98.0 (89.1–99.9)
New infections: n/N (%)	4/4 (100)	3/3 (100)

*PCR* polymerase chain reaction, *95* *% CI* 95 % confidence interval, *ETF* early treatment failure, *LCF* late clinical failure, *LPF* late parasitological failure, *ACPR* adequate clinical and parasitological response, * n/N number of new infections divided by number of samples analysed by PCR

### Clinical adverse events

ASAQ treatment was associated with asthenia, vomiting, pruritus, abdominal pain, and headache in a few patients, whereas few AL-treated children reported asthenia and vomiting. These adverse events occurred between day 1 and day 3 (Table 4). Asthenia was the most frequently reported adverse event in both groups, while ASAQ-treated children had more adverse events than AL-treated children (p = 0.004).

**Table 4:** Adverse events associated with artesunate-amodiaquine (ASAQ) and artemether-lumefantrine (AL) treatment in Owando health facilities

Adverse events	ASAQ (n/N; %)				AL (n/N; %)^a^		
	Day 1	Day 2	Day 3	Total	Day 1	Day 2	Total
Asthenia	4/61; 5.6	6/61; 9.8	3/59; 5.1	13/181; 7.2	1/57; 1.8	1/56; 1.8	2/113; 1.8
Vomiting	3/61; 4.9	0/61; 0	0/59; 0	3/181; 1.6	1/57; 1.8	0/56; 0	1/113; 0.9
Headache	0/61; 0	1/61; 1.6	0/59; 0	1/181; 0.6	0/57; 0	0/56; 0	0/113; 0
Urticaria	0/61; 0	1/61; 1.6	0/59; 0	1/181; 0.6	0/57; 0	0/56; 0	0/113; 0
Abdominal pain	1/61; 1.6	1/61; 1.6	1/59; 1.7	3/181; 1.7	0/57; 0	0/56; 0	0/113; 0
Total	8/61; 13.1	9/61; 14.8	4/59; 6.8	21/181; 11.6^†^	2/57; 0.7	1/56; 0.4	3/113: 2.7^†^

*N* number of patients followed up, *n* number of patients with adverse events

^a^There were no reported adverse events on day 3 in the AL group

^†^There was a statistically significant difference (p = 0.004) in the proportions of ASAQ- and AL-treated patients reporting adverse events

## Discussion

In 2006, the Republic of Congo adopted a new drug policy for malaria, which includes the treatment of uncomplicated malaria with ASAQ and AL, treatment of severe and complicated malaria with parenteral quinine, and recommendations for collective and individual preventive measures [13]. Long-lasting insecticide-treated nets (ITN) to protect pregnant women and children under 5 years old and intermittent preventive treatment during pregnancy (IPTp) with SP are the key measures of malaria prevention. The efficacy of ASAQ and AL had been assessed earlier in two sites, the rural Department of Pool and Brazzaville when the new anti-malarial drug policy was in preparation [14–16]. A longitudinal survey in urban and suburban health facilities in Brazzaville, the capital city, from 2003 to 2007 showed high percentage of clinical malaria episodes [10]. In 2011 and 2012, a study conducted among children under 5 years old and pregnant women in Brazzaville and Pointe-Noire (western Congo along the Atlantic coast) reported a reduction of malaria prevalence due to preventive measures [16]. This reduction in malaria burden could have been even greater if treatment for malaria had been provided free of charge to all malaria-infected patients in public health facilities since 2007, as initially planned by the government. Unfortunately, the limited supply of ACT in health centres and the high price of these drugs in pharmacies have limited the success of the measures [22].

RDTs are important tools that contribute to fight effectively against malaria. The ease of use of these devices allows untrained medical personnel to use them after a short training. Despite many advantages, the use of RDTs in Congolese health facilities is limited by its high cost and/or lack of adequate supply to meet the country’s demands. In public health centres with a microscope, the performance of microscopy to establish malaria diagnosis costs 500 FCFA (US $1.00). The local laboratory suppliers sell a box containing 25 RDTs for 25,000 FCFA, i.e. US $2.00 per RDT. At this price, RDT is not competitive in public health facilities.

In Uganda, the treatment of clinical malaria episodes was cost-effective when diagnosis was established with RDT, as compared to microscopic examination of thick blood films [23], while in Tanzania the benefit of RDT was observed in moderate and low transmission areas [24]. In Ghana, a reduction in treatment cost was obtained with RDT, as compared with presumptive treatment, but RDT was not advantageous when compared to microscopic diagnosis [25]. Several studies have shown that anti-malarial treatment is more cost-effective when diagnosis is confirmed with RDT, as compared to treatment based on presumptive diagnosis [26]. Although Global Fund provides funding to recipient countries to acquire RDT [27], the impact of this programme is not yet known. In several research studies conducted in sub-Saharan Africa, RDTs have been acquired as part of research projects funded by donors or as a donation **[**23–25, 28–31]. In African private hospitals, several trademarks of RDT are used without taking into account the evaluation of RDT performed by the WHO [32–34].

Under these conditions, microscopy is expected to continue to occupy an important place in the diagnosis of malaria in the context of declining malaria prevalence, with a decrease in the number of clinical malaria cases, and for determining parasite density, when required, as long as RDT is not subsidized like drugs. Whether malaria diagnosis is confirmed by microscopy or RDT, a strong commitment of sub-Saharan African states would be essential to implement the national and regional anti-malarial treatment guidelines [35].

This is the first epidemiological study conducted on malaria in the northern part of the country reporting malaria percentage in 0–11 years old febrile children attending health facilities spontaneously and drug efficacy. A reliable diagnosis was established in individual patients using both microscopy and RDT. Advantage Mal Card^®^ RDT is known for its good performance [33, 34], and it is the second time that this device was used in an epidemiological study in the Republic of Congo [17]. The combination of these two diagnostic methods ensures that these data can serve as a reference for future studies in the city of Owando.

The choice of age group 0–11 years constitutes one of the weaknesses of this study. An enrollment of all symptomatic malaria-infected patients (children and adults) would have generated more representative data in the health centres. The choice of 0–11 years old is justified by a limited quantity of available RDT. However, the 0–11 years age group is pertinent and of interest because it includes children under 5 years old who are considered as one of the target age groups, together with pregnant women, in the fight against malaria. The longitudinal study in Brazzaville from 2003 to 2007 (i.e. before the new anti-malarial drug policy based on ACT) had shown that in a suburban area of Brazzaville, 46.6 % of febrile children under 5 years old and 63.5 % of febrile patients aged between 5 and 10 years had clinical malaria [10]. By contrast, in the urban area of Brazzaville, 24 % of febrile patients under 5 years old and 33.5 % of patients aged between 5 and 10 years had malaria [10]. Studies conducted in Mayombe forest in southern Congo, where the annual entomological inoculation rate is high but variable (80–400 infective bites/man/year) [36], showed the frequency of malaria-associated clinical episodes of 35.4, 32.1, and 33.3 % in febrile children aged between 0 to <2 years, 2 to <5 years and 6–15 years, respectively [37]. This latter study also reported that malaria was the third cause of consultation in under 2 years old children and the fourth cause among children aged 2–15 years, after respiratory and gastro-intestinal diseases. With the low percentage of clinical malaria episodes among febrile children attending public health facilities in Owando city, it is likely that the majority of 0–11 years old patients consult for febrile diseases other than malaria as in Mayombe forest, including acute respiratory infections, upper respiratory tract infections, and gastro-intestinal infections.

Despite the unavailability of inoculation rate data, the city of Owando, located in a forest with shallows that are often flooded and scattered dwellings, may be considered as an area of high transmission encountered in Congolese forest regions. However, based on the results of the present study, the percentage of malaria-associated clinical episodes (i.e., 19.5 %) among patients 0–11 years attending Owando health centres appeared to be unusually low. Further studies at different times of the year are required to characterize malaria transmission in Owando.

In therapeutic efficacy studies, RDT which detects *P. falciparum* lactate dehydrogenase (PfLDH) can be used for rapid screening of patients for inclusion, and parasite density can be determined by microscopy [28]. In the present study, RDT was used to screen patients for inclusion in the study of ASAQ and AL efficacy. The PCR-corrected efficacy in Owando city was 100 % for ASAQ and 98.0 % for AL, which is in agreement with the results obtained in an earlier study conducted in a rural area in the Department of Pool, 200 km from Brazzaville, where the efficacy rate was 98.5 % for ASAQ (non-coformulated tablets) and 100 % for AL [13]. These ASAQ and AL drug formulations had 94.4 and 97.1 % efficacy, respectively, in previous studies in Brazzaville [15, 16]. Adverse events related to ASAQ intake and reported in studies in Congo [14–16] may become one of the causes of non-compliance among patients or their parents for this drug provided free of charge in public health facilities. AL is generally better accepted by the population, which has led the Congolese health authorities to recommend this ACT as the first-line drug and ASAQ as the second-line drug since 2014 [38]. However, AL requires six doses to cure malaria, and it is recommended to take AL with fatty food or drink. There are at present on the Congolese market more than a dozen AL specialties. Several dispersible formulations for children, with a fruity taste facilitating administration to children, are also available. The abundance of AL specialties available and freely prescribed in health facilities increases the risk of dissemination of poor quality drugs.

## Conclusion

This is the first study that provides data on clinical malaria episodes in patients aged between 0 and 11 years attending public health facilities in Owando, located in northern Republic of Congo, and clinical efficacy of two ACT (ASAQ and AL) for the treatment of uncomplicated falciparum malaria. Data presented here show a relatively low proportion of clinical episodes of malaria among febrile children attending public health facilities and high efficiency of both ACT recommended by the Congolese National Malaria Control Unit for the treatment of laboratory-confirmed uncomplicated malaria in health facilities.
